# Diacylglycerol kinase as a possible therapeutic target for neuronal diseases

**DOI:** 10.1186/1423-0127-21-28

**Published:** 2014-04-07

**Authors:** Yasuhito Shirai, Naoaki Saito

**Affiliations:** 1 Laboratory of Chemistry and Utilization of Animal Production Resources, Applied Chemistry in Bioscience Division, Graduate School of Agricultural Science, Kobe University, Rokkodai-cho 1-1, Nada-ku, 657-8501 Kobe, Japan; 2 Laboratory of Molecular Pharmacology, Biosignal Research Center, Kobe University, Rokkodai-cho 1-1, Nada-ku, 657-8501 Kobe, Japan

## Abstract

Diacylglycerol kinase (DGK) is a lipid kinase converting diacylglycerol to phosphatidic acid, and regulates many enzymes including protein kinase C, phosphatidylinositol 4-phosphate 5-kinase, and mTOR. To date, ten mammalian DGK subtypes have been cloned and divided into five groups, and they show subtype-specific tissue distribution. Therefore, each DGK subtype is thought to be involved in respective cellular responses by regulating balance of the two lipid messengers, diacylglycerol and phosphatidic acid. Indeed, the recent researches using DGK knockout mice have clearly demonstrated the importance of DGK in the immune system and its pathophysiological roles in heart and insulin resistance in diabetes. Especially, most subtypes show high expression in brain with subtype specific regional distribution, suggesting that each subtype has important and unique functions in brain. Recently, neuronal functions of some DGK subtypes have accumulated. Here, we introduce DGKs with their structural motifs, summarize the enzymatic properties and neuronal functions, and discuss the possibility of DGKs as a therapeutic target of the neuronal diseases.

## Introduction

Diacylglycerol kinase (DGK) is the enzyme which phosphorylates diacylglycerol (DG) resulting in the production of phosphatidic acid (PA) [1-5]. Both DG and PA are very important signaling molecules. DG regulates the activity and localization of several proteins, including protein kinase C (PKC), chimerins, Unc-13, and Ras guanyl nucleotide-releasing protein (RasGRP). PA also activates several enzymes, including phosphatidylinositol 4-phosphate 5-kinase, mammalian target of rapamycin (mTOR), and atypical isoforms of PKC [6-10]. Therefore, DGK is thought to be a key enzyme that regulates numerous cellular responses by regulating balance of the two lipid messengers.

**Figure 1:**
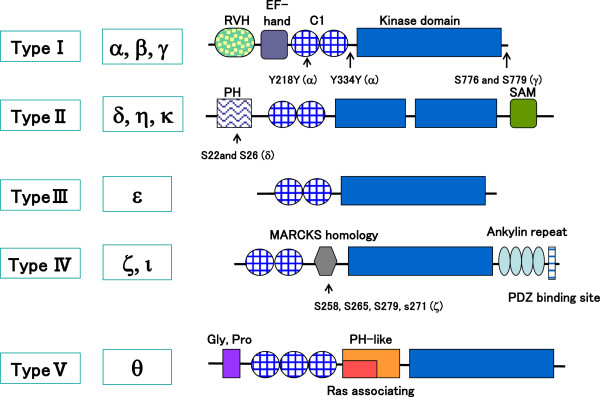
**Schematic illustration of DGKs with the phosphorylation sites.** RVH; recoverin homology domain; PH; pleckstrin homology, SAM; sterile alpha motif, MARCKS; myristoylated alanine rich protein kinase C substrate phosphorylation site. Numbers and alphabets show the phosphorylation sites reported.

So far, ten mammalian subtypes of DGK have been cloned [11-20] and categorized into five groups depending on their structural motifs (Figure 1). All DGKs have two cysteine-rich regions (C1A and C1B domains), except for DGKθ which has three regions, in the regulatory domain of the N-terminal half of the molecule. These C1 domains of DGKs are homologous to those of PKC, which shows a DG-dependent protein kinase activity. However, not all of the C1 domains but only those of DGKβ and γ show the binding activity to DG [21]. All DGKs have a catalytic domain in the C-terminal half of the molecule, and the catalytic domain of Type II DGKs (δ, η, and κ) is separated into two portions by an insertion. In addition to these domains, they have different structures depending on their groups. Type I DGKs, DGKα, β, and γ, have a recoverin homology (RVH) domain and an EF-hand motif. The RVH and EF-hand motif domains are thought to be a calcium ion sensor as described below. Type II DGKs, DGKδ, η, and κ have a pleckstrin homology (PH) domain at N-terminus and have sterile alpha motif (SAM) domain at C-terminus. Type III DGK, DGKϵ, has only the C1 domains. Type IV DGKs, DGK ζ and ι, have a myristoylated alanine rich protein kinase C substrate phosphorylation site like region (MARCKS homology domain) between the C1 and catalytic domains, four ankyrin repeats, and a PDZ binding site at C-terminus. Finally, Type V DGK, DGKθ has a proline and glycine rich domain as well as a PH domain overlapping with a Ras associating domain. In addition, many splice variants are reported [3,5].

Each subtype of DGK shows a subtype-specific tissue expression pattern (Table 1). For example, DGKβ, γ, ϵ, ζ, ι, and θ are localized in neurons and DGKα is reported to be localized in oligodendrocytes, although most DGKs are abundantly expressed in brain [22]. Specifically, DGKβ is expressed in caudate putamen, hippocampus, and cerebral cortex but not in cerebellum [12]. Instead, cerebellum expresses DGKγ, θ, and ζ. In addition, DGKα is highly enriched in immunological organs such as spleen and thymus. DGKζ is highly expressed in thymus and brain, with substantial levels in skeletal muscle, heart, and pancreas [16,23]. DGKδ shows ubiquitous expression including skeletal muscle and testis [15,24]. The different expression patterns suggest a subtype-specific function of DGKs. Indeed, DGKα and ζ knockout (KO) mice demonstrate their importance in the immune system [25,26] and DGKζ KO mice reveal its important role in cardiac hypertrophy [27]. Moreover, DGKδ plays a key role in insulin resistance in diabetes [28]. However, in spite of the abundant expression of DGKs in brain, their neuronal functions had been unknown for a long time. Recently, our and other groups have reported the importance of DGKs in brain functions [22,29-32]. In the following sections, we summarize the enzymatic properties and neuronal functions of DGKs for the development of drugs targeting the neuronal diseases.

**Table 1:** Characteristics of mammalian DGK subtypes

	**MW(KDa)**	**Localization**	**Enzymatic property**	**Function/Phenotype of KO**	**References**
α	80	thymus, spleen> > kidny, brain (oligodendrocytes)	Ca2+, PS dependent R59022 sensitive activated by PIP3, PI(3,4)P2	T cell anergy tumor invasion insulin release	[11,25,33-37]
β	90	brain(CP > hip, cortex, olf) > adrenal gland > small intestine	Ca2+, PS dependent activated by PI(4,5)P2	impairment of memory impairment of memory (mania-like) severe seizure	[12,29-31,38,39]
γ	88	retina, brain(Cb, hip)> > other tissues	Ca2+, PS dependent R59022 sensitive	insulin release	[13,38]
δ	130	skeletal muscle > testis, colon	Ca2+, PS independent	type II diabetes EGF signaling, seizure	[15,24,28,40,41]
η	130-140	testis > brain, lung, spleen > heart	Ca2+ insensitive	bipolar disorder (?), Ras-Raf-MEK signaling	[16,42-45]
κ	142	testis > spleen, pracenta,	Ca2+ insensitive	hypospadias	[20,24]
ϵ	64	retina,brain,testis > ovary> > skeletal muscle heart	Ca2 + independent selectively for arachidonoyl DG	seizure	[14,46]
ζ	104	thymus > brain(Cb, hip, olf)> > skeltal muscle, heart, pancreas	Ca2 + insensitive Mg2+, PS dependent	T cell anergy, cell cycle control spine maintenamce	[17,26,27,33,35,47,48]
ι	130	retina > brain (hip, CP, cortex, Cb, Dg)	Ca2+ insensitive	Ras GRP, Rap 1 signaling	[19,22,49,50]
θ	110	brain(Cb, hip)> > small intestine, liver etc.	Ca2+ insensitive	neurotransmitter release?	[18]

Cb; cerebellum, CP: caudate putamen, Dg; dandate gyrus, hip; hippocampus, Olf; olfactory bulb.

## Review

### Regulation of the enzymatic activity

Although the enzymatic characteristics of all DGK subtypes have not been investigated, the activities of some DGKs are regulated by ionic detergents and phospholipids [33,34]. For example, the activities of DGKα and ζ depend on deoxycholate or cholic acid, so that a detergent, octyl glucoside, is used for the DGK assay. But the dependency of phospholipids seems to be subtype specific (Table 1). The activity of purified DGKα is remarkably enhanced by phosphatidylcholine (PC) but not by phosphatidylinositol (PI), while DGKζ is activated by PI and phosphatidylserine (PS), but not so remarkably by PC [33]. DGKα is activated by PI3,4,5-trisphosphate (PIP_3_) and PI3,4-bisphosphate [PI(3,4)P_2_], while DGKβ is activated by PI4,5-bisphosphate [PI(4,5)P_2_] [34]. Moreover, divalent cations including calcium and magnesium are also required for activation of DGKα and ζ [35,51]. Specifically, Type I DGKs are believed to depend on calcium because they have the EF hand motif and RVH domains. The calcium dependency of DGKα has been clearly shown *in vitro*[35]. In addition to its role as a calcium sensor, the N-terminus region has an inhibitory effect on the kinase activity [52,53]. Indeed, the N-terminus region binds to its C-terminal region containing the kinase domain and C1 domain in the absence of calcium [54]. However, the *in vitro* calcium-dependent activity of DGKβ and γ has not been reported, although their EF-hands seem to bind to calcium and to regulate their kinase activity as well. Information of the enzymatic properties of each DGK subtype is not enough, and the precise enzymatic characterization should be carried out using the purified proteins under the respective optimized conditions.

All DGKs except for DGKβ are localized in cytoplasm in several cells, but some DGKs show the translocation to the plasma membrane and/or intracellular organelles in response to several stimulations [35,55-61]. For example, DGKγ is translocated from the cytoplasm to the plasma membrane by calcium, phorbol ester, and purinergic receptor stimulations [55]. DGKα is also translocated to the plasma membrane by calcium [35], purinergic receptor stimulation [55], and T-cell receptor stimulation [56]. Moreover, DGKδ translocates to the plasma membrane in response to phorbol ester [57]. These are coincident with the fact that DG is produced on the plasma membrane and DGKs work there. In addition to the plasma membrane, DGKα is accumulated at the Golgi complex in the case of arachidonic acid and vitamin E stimulations [55,58]. Furthermore, it is reported that DGKζ is translocated from the nucleus to the cytoplasm [59], and DGKθ and γ are localized in the nucleus [60,61], consistent with the fluctuation of DG and PA contents in the nucleus [62-64]. These findings indicate that translocation is the key regulation mechanism to define where and how long each DGK works, and suggest that DGKs are important for the DG/PA metabolism in the plasma membrane and many organelles including nucleus and Golgi complex. In the case of DGKβ, the plasma membrane localization seems to be a critical for its physiological function [29].

The translocation and activation of some DGKs are regulated by the phosphorylation reaction. We have revealed that DGKγ is subtype-specifically phosphorylated by PKCγ at Ser-776 and Ser-779 upon the purinergic stimulation, resulting in up-regulation of its lipid kinase activity [65]. The membrane translocation of DGKδ is regulated by the phosphorylation at Ser-22 and Ser-26 by conventional/classical PKC (cPKC) [66], and the nuclear export of DGKζ is dependent on the phosphorylation at the MARCKS homology domain by PKCα [67]. Not only the serine phosphorylation but also the tyrosine phosphorylation is important: the membrane translocation of DGKα is dependent on phosphorylation at Tyr-334 (Tyr-335 in mouse) by Src family tyrosine kinases [58,68,69]. We also found that Tyr-218 of DGKα is phosphorylated by c-Abl tyrosine kinase and the phosphorylation regulates serum-induced nuclear export of the enzyme [70]. These results indicate that the phosphorylation is one of the important regulation mechanisms for the spatial regulation of diacylglycerol signaling.

### DGK and brain function related to neuronal disease

#### DGKβ and η in mood disorder and memory loss

Most subtypes of DGKs are abundant in brain (Table 1 and Ref. 22). Above all, high levels mRNA of DGK β, γ, ζ, ι, and θ are detected in the neurons. *In situ* hybridization reveals that DGKβ is expressed in the caudate putamen, accumbens nucleus, and hippocampus [12]. Indeed, the protein expression of DGKβ in these regions is reported [29,38]. DGKβ is not present at the birth but its expression is rapidly increased from day 14 to day 28, and is localized on the plasma membrane of spines [38], suggesting its importance in the neuronal network. Indeed, Goto et al. have reported that DGKβ is expressed at post synaptic sites in medium spiny neurons constituting the striatonigral and striatopallidal pathways [39,71].

To investigate the neuronal functions of DGKβ, we produced its KO mice and found that the primary cultured hippocampal neurons from DGKβ KO mice had less branches and spines compared to the wild type. In addition, long-term potentiation in the hippocampal CA1 region of the DGKβ KO mice was reduced, causing impairment of cognitive functions including spatial and long-term memories in Y-maze and Morris water-maze tests [29]. Furthermore, the KO mice showed impairment of emotion [30]: DGKβ KO mice spent longer time in the center area in the open field test, and in the open arms in elevated plus maze test than the wild type. On the other hand, DGKβ KO mice showed normal input–output relationship and behavior in prepulse inhibition test and social interaction test [29,31]. These results suggest that DGKβ KO mice have a mania-like behavior with memory loss, although their social skill and basal synaptic function are normal. The importance of DGKβ in the memory and emotion fits to the localization of DGKβ in hippocampus and caudate putamen, and its developmental changes [38].

Interestingly, the impairment of emotion and memory of DGKβ KO mice is rescued with the lithium treatment for ten days ([30], and unpublished data). The effect of lithium seems to involve in GSK3β inhibition [30]. These results suggest that DGKβ can be a possible target of memory loss and mood disorder. Indeed, DGKβ is reported as one of the learning-regulated genes in a research of age-dependent memory impairment [72]. This report indicates that histone acetylation is associated with age-dependent memory impairment and histone deacetylase inhibitor is expected as a drug for memory loss [73]. Moreover, one of the splice variant forms of human DGKβ, which lacks 35 amino acids at the C-terminus but has an additional 4-amino-acid extension (DGKβ SV3; GenBank accession number AX032745), is associated with a human DGKβ EST that is annotated as differentially expressed in patients with mood disorders [74]. The splice variant form does not induce branches and spines [29], supporting the important role of DGKβ in spine formation.

Similarly, a mutation in DGKη is reported to correlate with bipolar disorder [42-44], of which mRNA seems to be concentrated in hipocaumpus and dendate gyrus, although the expression level of the subtype in the brain is lower than in testis and tumor-derived cells [45]. Baum et al. have reported strong association between bipolar disorder and SNPs located in a gene encoding DGKη by the genome-wide association study using the samples from European origin [42], and an increase in its mRNA level has been reported in some patients with bipolar disorder and schizophrenia [43,44], On the other hand, other studies have not confirmed the association [75-77]. The controversial results may be due to the races and methods employed. The precise experimental procedures would be necessary to prove the involvement of DGKη in bipolar disorder.

#### DGKζ and ι in spine modulation and neurotransmitter release

DGKζ, which is abundant in cerebellum, hippocampus, and olfactory bulb, is involved in spine maintenance [47,78]. For this function, its enzymatic activity and localization at the excitatory postsynaptic site by binding to some PSD95 family proteins through the PDZ-binding domain are critical. Interestingly, DGKζ is detected in the nucleus in the hippocampal neurons but it is translocated to the cytoplasm by ischemia or kainate stimulation [79,80], suggesting its role in the protection against ischemia. The subtype is also expected to be involved in the leptin receptor signaling because it associates with leptin [81]. However, detailed mechanisms are still unclear. DGKι also has the PDZ binding motif. The subtype is abundantly detected in hippocampus, dendate gyrus, and retina with moderate levels in the cortex, caudate-putamen, and thalamus [19,22,82]. However, the spine density and morphology of neurons in DGKι KO are normal [49]. Instead, DGKι regulates the presynaptic release during metabotropic glutamate receptor-dependent long-term potentiation [32,49]. Abnormal behaviors including impairment of memory and emotion of DGKζ and ι KO mice have not been reported yet.

#### DGKβ, ϵ, and δ in seizure

Seizure is a relatively common neuronal disease and at least three DGK subtypes are related to the disease. DGKϵ is uniformly expressed in brain, and its KO mice show an increased resistance to electroconvulsive and faster recovery than the wild type [46]. DGKδ is also associated to seizure, although its neuronal expression is very low: DGKδ gene is disrupted in a female patient with a *de novo* balanced translocation, who exhibits seizure with several dysfunctions, and electroencephalographic assessment of DGKδ mutant mice revealed abnormal epileptic discharges and electrographc seizures [40]. In addition, we also showed that seizure is severer in DGKβ KO mice than the wild type [31].

#### Other DGKs

DGKα is detected in oligodentrocytes, although the subtype is not enriched in brain [11,83]. DGKγ, which is predominantly localized in Purkinje cells and hippocampus, is present at birth and then gradually increased [13,38]. The mRNA expression of DGKθ is the highest in cerebellum and hippocampus [18] and it is suggested that DGKθ is involved in neurotransmitter release [84]. However, further examination would be necessary to understand physiological functions of DGKα, γ, and θ in brain.

### Perspective

As described above, some DGKs are important for neuronal functions. This is supported by their abundant and subtype-specific expression in brain. Generally, DGKs are likely involved in the spine formation and maintenance, contributing to higher brain function including memory and emotion. However, the detailed mechanisms of the DGK-mediated morphological change of spines and neurons are not clear. The membrane lipids including PA and DG seem to be the key molecules. Indeed, we found that mTOR and cPKC, which are activated by PA and DG respectively, are involved in the DGKβ-induced neurite induction and branching [unpublished data], and the kinase activity is necessary for the DGKζ-mediated spine maintenance [22,47]. Alternatively, PIP_2_ and/or PIP_3_ may be additional key lipids because some DGKs can be activated by these inositol phospholipids, which are related to actin cytoskeletal rearrangement and membrane traffic [85]. On the other hand, unknown mechanism like a kinase-independent pathway may additionally exist. For example, we have recently found that there is a kinase-independent pathway in the DGKβ-induced neurite induction and branching [86]. Further investigations are necessary to reveal the whole story regulating the shape of the neuronal membrane by DGKs.

The facts described in this review suggest that some DGKs can be a target for the therapy of neuronal diseases including memory loss, mood disorder, and seizure. To develop the drugs targeting DGKs for these neuronal diseases, more precise experiments should be performed using human patients. For example, there is still no evidence that splice variant forms of DGKβ is expressed at protein level in human patients of bipolar disorder, although it is suggested to association with mood disorders [74]. More importantly, it is necessary to find subtype-specific inhibitor and/or activator of each DGK subtype because DGKs have subtype-specific and numerous physiological functions as summarized in Table 1. Recently, Sakane et al. have developed a new DGK assay method suitable for the high throughput screening [87]. Sakane’s and our groups are collaborating to find specific inhibitor and/or activator of each DGK subtype. In near future, it will be possible to provide information about DGK-subtype specific inhibitor and/or activator which would be useful for the development of drugs targeting DGK for neuronal diseases.

DGKs are involved in not only neuronal diseases but also other diseases including diabetes, immuno-dysfunctions, and cardiovascular diseases. In addition, the correlation between cancer and DGK has been recently reported. For example, DGKα is involved in the progression of human hepatocellular carcinoma [88], and is necessary for the invasion of lung cancer [89]. These facts suggest that DGK can be a target of multiple diseases including diabetes, cancer, and neuronal diseases. Again, in addition to the comprehensive research to reveal subtype-specific functions DGKs, subtype-specific compounds would be necessary to develop drugs targeting DGKs without side effect.

## Conclusion

**Figure 2:**
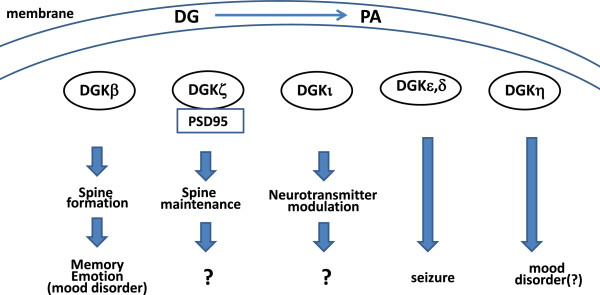
**A schema of functions of DGKs in brain.** DGKβ regulates spine formation and branching, contributing to higher brain functions including memory and emotion. DGKζ and ι are involved in spine maintenance and neurotransmitter modulation, respectively. DGKϵ and δ are reported to be related to mood disorder. DGKη seems to be correlated to seizure. The control of membrane DG and PA is a key step, and protein-protein interaction like DGKζ and PSD95 is critical for the functions of DGKs.

In conclusion, some DGKs are involved in formation and maintenance of spines through the control of membrane lipid and protein-protein interactions, contributing higher brain functions including memory and emotion (Figure 2). Thus, some DGKs can be therapeutic target for neuronal diseases including mood disorder, memory loss, and seizure.

## Competing interests

There is no financial and non-financial competing interests.

## Authors’ contributions

YS and NS conceived of the study and participated in its design and coordination and helped to draft the manuscript. All authors read and approved the final manuscript.
